# Introducing Patterns of Variability for Overcoming Compensatory Adaptation of the Immune System to Immunomodulatory Agents: A Novel Method for Improving Clinical Response to Anti-TNF Therapies

**DOI:** 10.3389/fimmu.2019.02726

**Published:** 2019-11-20

**Authors:** Tawfik Khoury, Yaron Ilan

**Affiliations:** ^1^Department of Gastroenterology, Galilee Medical Center, Nahariya, Israel; ^2^Faculty of Medicine in the Galilee, Bar-Ilan University, Safed, Israel; ^3^Department of Medicine, Hebrew University-Hadassah Medical Center, Jerusalem, Israel

**Keywords:** anti-TNF, rheumatoid arthritis, inflammatory bowel disease, loss of response, variability

## Abstract

Primary lack of response and secondary loss of response (LOR) are major obstacles to the use of anti–tumor necrosis factor (TNF)-based therapies in patients with rheumatoid arthritis or inflammatory bowel disease. Here, we review the mechanisms and methods for predicting LOR and the currently used methods for overcoming the ineffectiveness of anti-TNFs. The complex functions of TNF and anti-TNF antibodies, which can promote both pro- or anti-inflammatory actions, and the factors that affect the induction of immune tolerance to their effects are presented. The lack of rules and the continuous dynamics of the immune processes partly underlie the unpredictability of the response to anti-TNFs. Variability is inherent to biological systems, including immune processes, and intra/inter-patient variability has been described in the response to drugs. This variability is viewed as a compensatory adaptation mechanism of the immune system in response to drugs and may contribute to treatment LOR. Dose reductions and drug holidays have been tested in patients treated with anti-TNFs. Regular dose-based regimens may be incompatible with physiological variability, further contributing to treatment inefficacy. We present the concept of overcoming immune system adaptation to anti-TNFs by introducing patient-tailored patterns of variability to treatment regimens.

## Introduction

Anti–tumor necrosis factor (TNF) monoclonal antibodies (mAbs) are the most common biological drugs used for treating inflammatory disorders. Since the introduction of biological therapies almost 2 decades ago, specifically, the anti-TNFα agents, major alterations of the natural history of rheumatoid arthritis (RA) and inflammatory bowel disease (IBD) have been observed (1, 2). Anti-TNFα agents both induce and maintain clinical remission, improve quality of life, decrease the need for surgery, and improve morbidity, coupled with decreasing the total RA- and IBD-related costs (3).

Despite major progress and the wide use of these drugs, only a portion of treated patients achieve long-term clinical remission. Some patients who receive anti-TNFs fail to respond (primary failure), and others have loss of response (LOR) following an initial response (secondary failure) (4). Both primary and secondary failure are major obstacles to the long-term use of anti-TNFs in RA and IBD. Better understanding of the mechanisms for the development of drug resistance may enable improved responses (5). In the present review, we discuss some of the potential mechanisms for the compensatory adaptation of the immune system toward anti-TNF–based drugs, focusing on potential methods for overcoming it.

## Primary and Secondary Non-responsiveness to Anti-TNF Agents in RA and IBD

It is currently estimated that only up to 60% of patients with RA achieve long-term response to anti-TNF drugs (6, 7). One-third of patients with RA show inadequate primary response these medications (8). In a study of 157 patients treated with various anti-TNF formulations, 21% of the patients achieved 1-year clinical remission, and 58% of patients had >1.2 reduction of disease activity score (DAS28). There was moderate response according to European League Against Rheumatism (EULAR) criteria in 46% of patients, and 35% of patients had a good response (9). Primary failure was attributed to disease heterogeneity in terms of the types of inflammatory mechanisms and subsets of cells involved (10, 11).

In RA, there is no consensus for the definition of secondary failure, when efficacy is lost over time despite a good initial response. Secondary failure is considered if there is an increase in DAS28 of >0.6 during the previous 6 months or an increase in EULAR response (5, 12, 13). The time to discontinuation of a biological drug, or drug survival, is affected by loss of efficacy, immunogenicity, adverse events, and/or poor adherence. Loss of efficacy is the major cause of treatment discontinuation, and occurs in 48% of patients; 34% of patients experience adverse events (14, 15). Anti-TNF drug survival in patients with RA is 47 months (14). The overall 10-year retention rate of first-line anti-TNF agents is 23% (5, 16).

Primary LOR in IBD is defined as failure to achieve clinical remission as evaluated by clinical scores, including the Crohn's disease activity index (CDAI) and Harvey-Bradshaw index (HBI), and laboratory remission as evaluated by serum inflammatory markers. The timeframe within which primary response or non-response is determined varies between trials (17, 18). Nonetheless, expert consensus and clinical trials indicate that primary non-response to anti-TNF drugs should not be assessed prior to 14 weeks of infliximab (IFX) therapy or prior to 12 weeks of adalimumab (ADM) therapy (19). Moreover, in the clinical practice of some experts, patients are considered to have primary non-response after 6 months of anti-TNF treatment without evidence of remission. Secondary LOR in IBD is defined by lost or attenuated clinical and endoscopic response over time to anti-TNFs after an initial response to anti-TNFs. To confirm the diagnosis of secondary LOR, the patient must fulfill two conditions: develop reappearance of the clinical symptoms associated with disease exacerbation, and that the symptoms are mediated by inflammatory disease exacerbation of the underlying IBD (20). Primary failure of anti-TNF induction therapy occurs in up to 40% of patients with IBD in clinical trials and in 10–20% of patients in clinical series (21). Secondary LOR was reported in 25–61% of patients on anti-TNF maintenance therapy (22–24). A recent controlled study showed that >50% of patients with CD treated with IFX and ADM developed LOR (24). Additional trials have reported secondary LOR in 23–46% of patients 12 months after drug initiation. A review of 86 trials on patients with CD reported LOR incidence of 8–71%. The incidence of LOR with a median follow-up of 1-year was 33%. The rate of LOR in patients treated with IFX, ADM, and certolizumab pegol was 33, 30, and 41%, respectively. Overall, the mean percentage of LOR to anti-TNFs was 38%, with an annual rate of 20% per patient-year (25).

Taken together, both primary and secondary failure remain major obstacles to achieving a prolonged, sustainable effect of anti-TNFs in both RA and IBD.

## Difficulties in Predicting LOR to Anti-TNFs in RA and IBD Prevent Therapy Guidance in the Majority of Patients

Identifying the causes and biomarkers for predicting efficacy and for anticipating the development of primary or secondary LOR is an unmet need. It may also provide a means for selecting newer lines of therapy and minimizing adverse effects and cost. The causes of secondary loss of efficacy are not fully understood.

In patients with IBD, the underlying mechanisms of loss of effect include longer disease duration, smoking, and several genetic mutations (21). The data are still limited, and data on the role of these measurements in guiding therapy are conflicting (21). Those mechanisms are classified into several domains:

- Drug factors: Immunogenicity is defined by the formation of ADAbs in the setting of low biological drug levels. Both primary and secondary LOR are attributed to ADAbs. ADAbs neutralize the anti-TNF drug connecting to the Fab segment of the protein, or may bind solely to the anti-TNF molecule, promoting the formation of immune complexes leading to increased drug clearance through the reticuloendothelial system. ADAbs or sub-therapeutic trough concentrations explain LOR in only a proportion of patients with IBD, as many patients experience disease exacerbation with LOR in the setting of therapeutic drug concentrations and the absence of ADAbs (26). Combo-therapy with immunosuppressive and immunomodulatory drugs aimed at suppressing the reaction of antibody formation has not been consistently shown to improve treatment durability or efficacy in all studies. Additionally, less immunogenic humanized anti-TNF therapies have similar rates of LOR as the chimeric IFX (20, 27). The current evidence supporting the routine estimation of ADAbs or serum trough levels during anti-TNF therapy is limited; however, they are suggested as guidance for changing between therapeutic biologics in secondary failure (5).
- Pharmacokinetic failure is defined by decreased levels of the drug with typically absent or low anti-drug antibodies (ADAbs). The pathogenesis is secondary to accelerated non-immune drug degradation via tissue or systemic circulation. The three main mechanisms underlying pharmacokinetic failure are proteolytic catabolism within the reticuloendothelial system, mAb binding to Fc gamma receptors, and degradation in lysosomes by binding to membrane-bound TNF (28–30).
- Autoantibodies: The presence of autoantibodies, including antinuclear antibodies (ANA) and antibodies against double-stranded DNA (anti-dsDNA), may contribute to LOR. Higher levels of these autoantibodies may interact with the anti-TNFs, reducing their efficacy (31).
- Genes and proteins expression: Alteration of the expression of the apolipoprotein (*APO*) genes, mainly *APO4*, which produces a protein with antioxidant ability, has been associated with LOR (32, 33). In patients with IBD, LOR cannot be attributed directly to pathways that bypass the action or induce resistance to anti-TNF therapy. An RNA microarray study showed that patients with LOR had elevated colonic expression of the pro-inflammatory chemokines CXCL20, CXCL9 (C-X-C motif chemokine ligand 9), and CXCL10. Patients with continued inflammation had elevated MMP3 (matrix metalloproteinase 3), MMP1, and MMP12. Patients with LOR had dysregulated cysteine and methionine metabolism pathways, implying alterations in the oxidative stress burden (32).
- Patients and disease phenotypes: Factors predictive of longer time to failure include obesity, smoking, higher baseline serum albumin, male sex, and thiopurine co-therapy. Higher baseline fecal calprotectin is associated with shorter time to failure (21, 34, 35). Elevated body mass index (BMI) is associated with poorer response to IFX and correlates with higher drug levels, but not a higher response rate, suggesting that circulating drug levels do not correlate with tissue levels (36).
- Fibrostenotic disease behavior has been associated with both primary and secondary LOR, and in those cases, surgical resection is more appropriate than biological therapy. Lower response rates have been described in fibrostenotic disease (37). Severe inflammatory activity has been associated with lower efficacy of anti-TNFs due to non-immune clearance of the drug, accounting for both primary and secondary LOR (38, 39). The proposed underlying mechanism for this is fecal loss of anti-TNFs through the ulcerated and sloughed colonic mucosa (40).
- Treatment factors: The dosing regimen is important for primary non-response. Remission at 4 weeks in patients receiving ADM was associated with a higher drug dose (41). A similar study on IFX (ACCENT 1) reported a lower primary non-response rate in patients who received a higher dose of the drug (17).
- Combo-therapy: A previous study (SONIC) showed that early co-treatment of IFX with immune modulators (azathioprine) vs. monotherapy had a higher response rate, accompanied by a significantly higher rate of mucosal healing. However, no similar data have been reported for ADM (42).
- Oxidative stress can dysregulate the cysteine and methionine pathways in patients with IBD with LOR. Both pathways are important for producing nicotinamide adenine dinucleotide phosphate (NADPH) and S-adenosylmethionine (SAM), which regulate oxidative stress by producing oxidative stress protein scavengers (32).

In patients with RA, most biomarkers used have insufficiently strong predictive value for predicting treatment response in individual patients with RA (43). Many baseline disease characteristics fail to predict the outcome, suggesting that drug metabolism or receptor adaptation may be contributing factors (44).

- Genotypes: Patients with RA with a TNF-308 G/G genotype, human immunoglobulin (Ig) allotypes in the IgG1 heavy chain (G1m1 and G1m17), and HLA (human leukocyte antigen)-DRB1 locus have better response (45–47). Five tagging single-nucleotide polymorphisms (SNPs) in the TNFRSF1B (TNF receptor superfamily member 1B) gene were studied in 1412 patients with RA, and the authors reported that carriers of the rs3397C/C, rs1061622G/G, and rs1061631A/A genotypes have increased risk for worse response to anti-TNFs. However, the association with specific SNPs only reached marginal significance and was not confirmed in a meta-analysis. Overall, these data do not support a major effect of TNFRSF1B variants in determining the response to anti-TNF drugs (48). SNPs in the steroid hormone–related genes showed significant correlation of CYP3A4 (cytochrome P450 family 3 subfamily A member 4) rs11773597 and CYP2C9 rs1799853, with changes in DAS28 after the administration of anti-TNFs. A model comprising eight steroid hormone–related variants predicted drug response (49). A review of all studies reporting associations between genetic variants in RA identified 25 SNPs as being associated with anti-TNF response. These were mapped to genes involved in T cell function, nuclear factor kappa B (NFκB), and the TNF signaling pathways (50). A genome-wide association study (GWAS) conducted in 372 patients with RA showed an association between the MED15 (mediator complex subunit 15) gene and the response to ETA (51). The impact of dose titration based on pharmacoeconomics in clinical practice remains questionable (52).
- Anti-drug antibodies: Most anti-TNF agents induce a certain degree of immunity, and ADAbs may limit drug survival (5, 53). It is unclear whether these antibodies are a major cause of the loss of anti-TNF clinical efficacy (5, 54). IFX is a chimeric mAb and is more immunogenic, but ADAbs also bind to the idiotype of the fully human mAb ADM. Etanercept (ETA) is associated with reduced immunogenicity (55). ADAbs were detected in the sera of 7–53 and 1–31% of IFX- and ADM-treated patients with RA, respectively, and were suggested to correlate with decreased response and increased adverse events (56, 57). The detection of ADAbs is confounded by the detection method used, high serum concentrations of rheumatoid factor, and the presence of the drug itself (58). In some studies, ADAbs were associated with reduced clinical response in RA, suggesting that monitoring drug levels may aid in optimizing the dosing regimen (59–61).
- Patients and disease phenotypes: In a study where 42% of patients stopped therapy, increased likelihood of discontinuation was associated with higher physician global scores and RA Disease Activity Index scores 6 months prior to stopping the TNF inhibitor, and a higher number of TNF inhibitors used previously. There was a lower percentage of ETA discontinuation than IFX and ADM (62). A study of 299 patients with RA reported that age, female sex, and high values of both disease activity and disability were predictors of non-response (63).
- Immune background: The presence of rheumatoid factor or anti-cyclic citrullinated peptide antibodies was associated with reduced response (64). Baseline serum levels of interleukin-6 (IL-6) predicted depletion of the drug and were suggested as a biomarker of treatment failure (65). Serum calprotectin had moderate predictive value for clinical response to anti-TNFs (66).

Overall, the currently available tests do not provide a valid tool for therapy guidance in terms of predicting primary and secondary failure.

## Current Methods for Overcoming Ineffectiveness of Anti-TNFs in RA and IBD Fail to Overcome LOR in the Majority of Patients

In RA, concomitant administration of immunosuppressive agents is commonly used for improving response rates to anti-TNFs. Improved results were noted in patients treated with methotrexate (MTX) in combination with anti-TNFs. The synergy between anti-TNF and MTX is not fully understood and can only be partially explained by suppression of ADAb formation and increased trough concentrations (5, 67–69).

Switching between different anti-TNF formulations is another commonly suggested method for improved response in RA and has been successful in some studies (70, 71). The improved response following switching is attributed to differences in structure, immunological action, immunogenicity, and pharmacokinetics (72). Switching was beneficial in secondary lack of effectiveness [defined as loss of ACR50 (American College of Rheumatology response criteria−50% improvement)] in 479 patients with RA. In these patients, the disease activity parameters improved from baseline upon use of IFX or ADM, but had increased prior to the switch. Switching from ETA to ADM restored the response achieved with the first drug. Several activity parameters that had improved from baseline upon use of ETA were maintained but were not improved further after switching to ADM. When switching due to adverse events, the second agent achieved a similar degree of response to that of the first agent (73). In a study of 356 patients with RA, 38 switched from IFX/ADM to ETA, 26 from ETA to IFX/ADM, and eight from one mAb (IFX/ADM) to another. Switches occurred due to primary failure (36.1%), escape (33.3%), or intolerance (30.6%). More switchers responded to the second anti-TNF regardless of molecules switched. The second anti-TNF had longer survival with the switch from a mAb to a soluble receptor than vice versa (74). Taken together these data support the notion that LOR may be improved by a switching strategy.

In a study of 99 patients with RA, switching took place if no reduction >0.6 in the initial DAS28 occurred after 12–24 weeks (inadequate response) or if a severe adverse event was reported. Switching was performed in 39% of patients. The retention of the first agent was 60%, and the mean time to switching was 14 months. After switching, there was a tendency toward decreased DAS28, and 43% of patients had good/moderate EULAR response; however, there was a low likelihood of remission and no significant improvement in functional capacity (75). In a trial of 300 patients with RA with persistent disease activity [DAS28–erythrocyte sedimentation rate (DAS28-ESR) ≥ 3.2] and insufficient response to anti-TNF therapy, patients were randomly assigned to receive a non-TNF targeted biologic agent or to switch to another anti-TNF. Within 6 months, 69% of patients in the non-TNF group and 52% in the second anti-TNF group achieved good or moderate EULAR response, and the non-TNF group had lower DAS28-ESR than the second anti-TNF group. At weeks 24 and 52, more patients in the non-TNF group vs. the second anti-TNF group showed low disease activity. The data suggest that a non-TNF biologic agent is more effective than a second anti-TNF for achieving good or moderate response at 24 weeks (8).

Several of these methods are also being used for overcoming LOR in patients with IBD. In CD, dose-optimization strategies for IFX using induction doses at 0, 2, and 6 weeks, followed by maintenance administration every 8 weeks, conferred better protection against ADAb formation (76). A randomized, controlled study of 69 patients with CD with secondary IFX failure showed that using an algorithm based on combined IFX and IFX antibody measurements reduced the average treatment cost per patient without negative effects on efficacy (77).

Re-induction is an effective strategy in LOR (35). Dose intensification was proposed as a means of overcoming LOR in IBD. Dose intensification with a median follow-up of 1 year was needed in 38% of patients for IFX, 36% for ADM, and 2% for certolizumab pegol. A mean 23% of patients needed anti-TNF dose escalation, with an annual risk of 18% (25). Following dose escalation for ADM-treated patients with CD, a clinical response was observed in 79 and 61% of patients at 3 months and 12 months, respectively (78). Compared with empirical adjustment, an algorithm for dose intensification and therapeutic drug monitoring of IFX trough levels and ADAb assays resulted in fewer dose escalations, i.e., 45 vs. 71%, without loss of efficacy (79).

Dose intensification of anti-TNFs is mainly used in the setting of secondary LOR, where there is a sub-therapeutic level of the drug and low/undetectable ADAb levels. It can be performed by either shortening the interval frequency or increasing the dosage. The efficacy of this strategy has been proven even without drug monitoring (80, 81). However, further studies have shown that drug level monitoring during dose intensification is more cost-effective and may reflect the recapturing response for anti-TNFs in patients who achieve an increment in drug level following dose intensification (82, 83). Implementing dose intensification in the presence of ADAbs has not been established. Dose intensification of IFX in the presence of ADAbs was associated with a paradoxical decreased response (84). Low ADA levels with detectable ADAbs were associated with drug failure (26). Patients with IFX ADAbs were more likely to fail dose intensification (82). Higher ADAb levels identify patients who do not respond to increased drug dosage (85). ADAbs are associated with lower ADA serum levels and a lower likelihood of remission. However, patients have experienced loss of ADAbs to ADA following dose escalation (86). IFX intensification in secondary LOR improved the clinical response while decreasing ADAbs irrespective of the levels of serum IFX and ADAbs (87). Increased serum IFX levels after dose intensification were associated with improved clinical outcomes and undetectable IFX ADAbs (88). Recent treatment algorithms suggest that dose intensification may overcome low ADAb levels (30, 80, 82, 89, 90).

Several studies suggested that combining immunomodulatory agents with anti-TNFs is can be used in IBD. The addition of immune modulators has mainly been implicated in immunogenicity-mediated primary LOR, which is defined by the inability of anti-TNFs to bind to the TNF molecules, resulting in increased immune-mediated drug clearance (80). Concomitant combo-therapy with an immunomodulator is used to prevent immunogenicity. Adding thiopurine or MTX as an immune modulator starting together upon the initiation of anti-TNF has been associated with decreased ADAbs formation (91) and can improve the clinical and histological outcomes, coupled with increased rates of steroid-free remission and decreased need for switching (19, 42, 92, 93). Notably, no difference in adverse effects, including infection and malignancy, were noted when combo-therapy was used as compared to biological monotherapy in one study (94).

Additional trials raised concern about the long term efficacy and safety of a combination therapy. Up to 45% of IBD patients who experienced LOR during a follow-up period of up to 8.5 years were followed using combination therapy with an immunomodulatory drug (59%) or monotherapy (40%). The median time to LOR was not different between groups. The data suggest that patients treated with anti-TNF monotherapy have similar LOR rates as patients on anti-TNF combination therapy (95). Switching to another anti-TNF may aid 50% of patients with IBD. Switching from ADM to IFX was beneficial in patients with LOR and in patients with undetectable ADM trough levels. The majority of patients required IFX therapy intensification during their first year of treatment (96). Recent trials have raised safety concerns, including comorbid malignant diseases such as lymphoma, with the concomitant use of other immunosuppressive drugs or increased dosages (97). A concomitant elemental diet (ED) with ADM in patients with CD showed that the ED group had a higher cumulative non-ADA LOR rate. ED reduced ADA LOR in IFX-intolerant or -refractory patients than in anti-TNFα-naïve patients. The ED group had lower serum TNFα levels (98).

None of the measures used for overcoming LOR are personalized, nor do they fit the dynamic type of the compensatory adaptations to anti-TNF therapy, which may change over time between patients and in the same patient. While they provide a solution for some patients, none can provide a prolonged response for the majority of patients.

## The Paradoxical Function and Tolerance Toward Anti-TNF Antibodies are Unpredictable and Dynamic Over Time

The mechanisms of action of both TNF and anti-TNF mAbs are not fully elucidated. The complex responses of the immune system to anti-TNFs, impact both their short- and long-term clinical effects. Many of these effects are dynamic and may occur over time, and vary between patients and in the same patient, making them irregular and difficult to predict.

Humans may develop tolerance of anti-TNFs, improving the response by reducing ADAb levels. Alterations of treatment regimens, where IFX is administered at week 0, 2, 6, and 14, and every 8 weeks thereafter, was associated with higher trough levels reducing ADAb development (99), supporting high-dose tolerance, which is induced by the high antigenic load (5, 100).

Both linear and non-linear eliminations have been reported for anti-TNF mAbs depending on the amount of the target antigen, immune reactions to the antibody, and patient demographics (28). Their clearance demonstrated non-linear kinetics due to receptor loss following repeated doses, which was proposed to be associated with disease severity (28, 29). Due to their molecular size, mAb distribution to tissues is slow, and their distribution volumes are low. Anti-TNFs are metabolized by phagocytes or by their target cells to peptides and amino acids, and are protected from degradation by binding to the neonatal Fc receptor (FcRn), which explains their long elimination half-lives.

TNF exerts both pro-inflammatory and immune-suppressive effects. Lower or higher TNF production characterizes many autoimmune diseases. TNF blocking in autoimmune and chronic inflammatory diseases is associated with unpredictable outcomes (101). Treatment timing and duration can alter this unpredictability. Both IFX, ETA, and ADM neutralize soluble TNF and bind to transmembrane TNF (tmTNF). They are dual-function and can act as antagonists by blocking TNF interactions with the TNF receptors TNFR1 and TNFR2, or initiate a reverse signaling cascade leading to apoptosis, cell activation, or cytokine suppression (55).

A paradoxical expansion of T helper 1 (TH1) and TH17 pro-inflammatory lymphocytes following IFX treatment may be another mechanism of LOR in some patients (102). Anti-TNF therapy is associated with drug-induced anti-dsDNA production and with the development of the manifestations of lupus and neuroinflammatory diseases (103). In patients with multiple sclerosis, anti-TNF treatment was associated with immune activation and disease exacerbation (104). The heterogeneity of TNFR usage during immunosuppression vs. the inflammatory tissue damage may underlie some of these findings. It implies that the effect of anti-TNF at receptor level is of greater relevance in human chronic inflammatory and autoimmune conditions (101). These paradoxical effects are unpredictable and dynamic over time.

Tolerance to TNF has been described at receptor level. Soluble TNFR1 (sTNFRI) is an endogenous mechanism for reducing serum TNF. Endotoxin tolerance via lipopolysaccharide (LPS)-preconditioning downregulates pro-inflammatory cytokine production. Tolerance mechanisms upregulates TNFRI, which binds and clears TNF while reversing the TNF-to-sTNFRI ratio (105, 106). tmTNF is transiently expressed on the surface of LPS-stimulated monocytes, macrophages, and dendritic cells, and can be enhanced following treatment with a TNF inhibitor (107).

Repetitive administration of low doses of human TNF to mice induces tolerance to the effects of mouse TNF via post-receptor mechanisms (108). No differences in pharmacokinetic parameters were noted in tolerant vs. control mice. There was an antibody response to human TNF, but the antibodies did not neutralize the mouse TNF. The tolerance did not protect mice against lethality induced by TNF. When tolerance was induced in athymic nude mice, which lack an antibody response, there were no effects on the levels of soluble receptors or receptor binding in the tolerant vs. control groups (108).

Overall these complexities further contribute to long term loss of effects of these drugs.

## Variability is Inherent to Biological Systems and Comprises Marked Intra/Inter-Patient Variability in Response to Drugs

Both intra- and inter-subject biological variability (BV) in biological and immune systems has been described at cellular organelle level, as well as at whole-organ level (109–115). This inherent variability is difficult to overcome. Lymphocyte subpopulation phenotype variability has been described when tested as biomarkers of immune-associated disorders. The antibody response toward pathogens includes expansion of antigen-specific B cells that is based on stochastic competition between competing cell fates, or deterministic cell fate decisions that execute a predictable program (116). Variability was noted for both cell proliferation and death decisions and evolved from heterogeneity in founder cells. The data imply that a small number of genetically identical founders are associated with the majority of the responses. A high rate of variability in the generation of CD4+ T regulatory cells (Tregs) is a major obstacle for cell therapy of immune-mediated disorders (117). An *ex vivo* cytokine release test, measured after stimulation of whole blood with various stimuli, showed high intra-group and inter-individual variability. The median coefficient of variation of the repeated tests was 29 and 52% for IL-1β and IL-8, respectively. Upon stimulation with endotoxin, a confidence interval of 60–140 and 70–271% was calculated for IL-1β and IL-8, respectively (118).

The inter- and intra-individual variability described in the response toward drugs has been attributed partly to pharmacogenomics- and pharmacodynamics-based drug metabolism, and drug responsiveness (119–122). However, there is heterogeneity between individual cells in their response to drugs (123). Complex physiochemical determinants of drug-target interactions in a cell have been described and are not defined by simple diffusion and intrinsic chemical reactions. The non-specific interactions of drugs and macromolecules in cells are beyond “simple” pharmacodynamics, affect drug function, and are difficult to control for. Non-specific interactions greatly slow the incorporation kinetics of DNA-binding drugs and have been attributed to anomalous drug diffusion in cells (123). Differential cell compartment effects affect intracellular drug kinetics variability (123). There is marked intra-patient variability in drug serum levels between days, suggesting additional underlying mechanisms (122, 124).

The inherent variability in biological systems evolves along a trajectory associated with the body's response to multiple internal and external triggers, and are aimed at reaching a newer steady state. These systems function under unpredictable conditions, are highly dynamic, and are therefore difficult to alter. Each exogenous trigger, e.g., anti-TNF antibodies, induces a compensatory adaptation mechanism that may lead to a paradoxical response, tolerance, and a new steady state.

## Dose Alterations and Introducing Variability into Anti-TNF Therapies is Associated With Improved Response

The high rate of LOR to anti-TNFs, along with their complicated mechanism of action at receptor/post-receptor level, has led to additional approaches for overcoming LOR. Both anti-TNF dosage escalations and reductions are used in the real-world setting. Intermittent dosing with drug holidays has clinical benefits while minimizing drug exposure and potential adverse effects (125).

Anti-TNF re-induction following a drug holiday has been suggested as a means of overcoming LOR. The outcome of this approach depends on the circumstances during which the drug holiday is commenced (21). Dose modifications compared to basal dose have been described in 7% of patients on ETA, 30% of patients receiving ADM, and 21% of patients on IFX. ADM and IFX have been associated with higher risk of dose escalation relative to ETA, and dose reductions are similar among all anti-TNFs (126).

Dose reduction schedules of anti-TNF as maintenance therapy in patients with spondylarthritis are used in clinical practice (127). Dose reduction implemented empirically for several years has improved treatment efficiency in RA (128). In a study of 153 patients, 45% received a lower dose after achieving remission or low activity at standard doses, and maintained good disease control. Dose titration of anti-TNF in RA by 67% of patients was not associated with a change in DAS28, and no patient dropped out because of disease worsening (129). An anti-TNF dose-tapering strategy was evaluated in patients with ankylosing spondylitis (AS). In the reduced dosing group, the median dose of anti-TNF corresponded to 0.67 of the initiated dose, and was 0.5 at 12 months. Up to 79% of patients did not require return to standard dosing regimen. Patients that had received reduced or standard dosing had similar mean change per year in the Bath AS Activity Index, C-reactive protein, Health Assessment Questionnaire Disability Index, Bath AS Functional Index, and quality-adjusted life-year (130).

In a prospective trial, 80 patients with CD and ulcerative colitis (UC) in clinical remission receiving IFX maintenance treatment were randomized to receive IFX dosing guided by a pharmacokinetic model, aiming to maintain a drug level using a (de-)escalation dashboard or to continue regular dosing. There was loss of clinical response in 36% of controls vs. only 13% of patients in the intervention group. In the intervention group, 50% had dose reduction while 35% had dose escalation. The clinical and laboratory benefits were achieved irrespective of the lack of change in drug level, and with narrowed dose range variability (131). The results support the premise that even simple dose alterations are associated with significant clinical improvement compared with regular fixed dosing.

## Overcoming Immune System Adaptation to Anti-TNFs by Introducing Patient-Tailored Patterns into Treatment Regimens

The unpredictability of the response to anti-TNF–based therapies, high LOR rate, and paradoxical activation of the immune system, along with empirical real-world data on the beneficial effects of drug holidays and dose reductions, supports evaluating BV as a method for overcoming LOR. Part of this inter- and intra-patient irregular behavior is viewed as normal adaptation attempts of the immune system in response to triggers such as the administration of anti-TNF.

**Figure 1:**
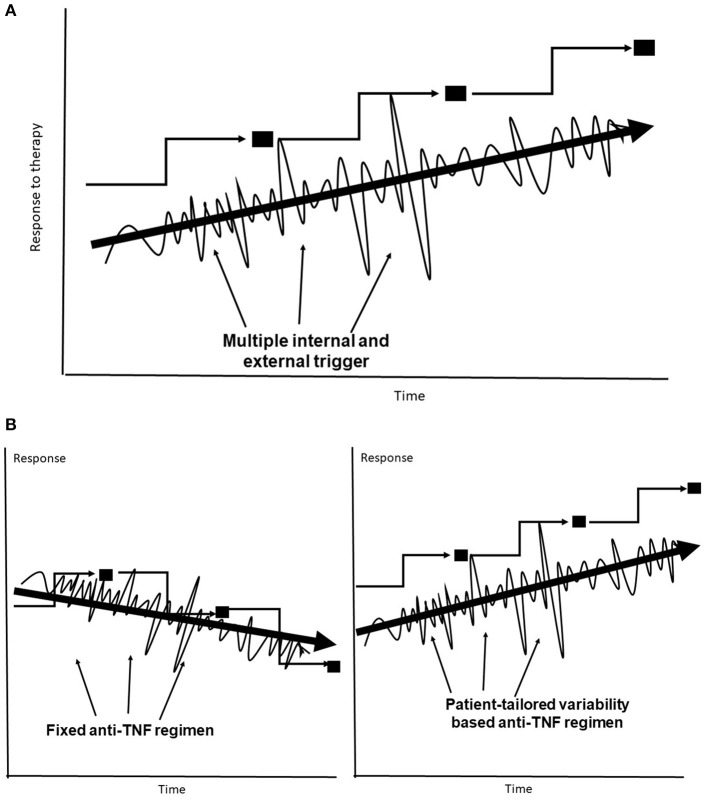
**(A)** The body's trajectory of compensatory adaptation in response to multiple internal and external triggers uses variability to reach a new steady state. **(B)** Fixed dosing may sometimes jeopardize the response to anti-TNF–based therapies, leading to lower steady states. Subject-tailored patterns of variability are introduced into anti-TNF administration along the trajectory for achieving an improved steady state.

Many biological systems lack fixed rules that remain constant over time. These systems are dynamic in both health and disease, as they are required to continuously respond to ongoing internal and external triggers in an attempt to reach a new steady state (132–135). The lack of rules in biological systems (132, 133), and the continuous dynamics of the immune processes (134, 135), along with the lack of understanding of some of existing rules, while responding to trigger(s) may underlie part of the unpredictability of the response to anti-TNFs. It has been proposed that the optimal state in variability is a U shape between a chaotic pattern of variability in a steady state and full predictability in a normal biological system (109, 110, 136–139). The body functions along a trajectory that implements variability patterns in an attempt to identify the optimal response to different triggers, including those by anti-TNF therapies. This behavior has an inherent variability that may not necessarily move toward a better point, makes mistakes, and can result in LOR (Figure 1).

The adaptation of the immune system may occur within a short time of drug administration, leading to primary failure, or following longer treatment periods, resulting in partial or complete loss of efficacy. The adaptation may manifest as immune tolerance in terms of lack of response to changes induced by the mAb at TNFR or post-receptor level. The inherent heterogeneity of the immune system response may result from the gradual accumulation of small amounts of intrinsic noise, which occur, for example, during cell differentiation (116).

Anti-TNF dosing using regular fixed regimens may not be compatible with the physiological variability in the immune system and may further contribute to LOR (140, 141). Fixed regimens may be incompatible with the random nature of the trajectories associated with the immune system, which both underlie inflammation and the compensatory mechanism for anti-TNFs. It has been proposed that, for various systems, the dynamic properties of the system may be associated with its evolution into a structure that optimizes their function (142). Therefore, even if there are rules, they may change constantly over time.

Interdependency between different network properties, which is applicable to many immune processes, many of which behave randomly, can be quantified. The dynamic systems theory suggests that biological systems are self-organized according to environmental, biochemical, and morphological constraints to find the most balanced state (143). It has been proposed that a patient-tailored variable regimen can overcome this adaptation, thereby improving the short- and long-term responses to anti-TNFs.

It has been proposed that the system's degree of variability requires augmentation to improve anti-TNF efficacy. Introducing greater variability into the system follows the same trajectory used by the body in its response to the triggers induced by the drug itself. This is expected to improve the response to anti-TNF mAbs under conditions of unpredictability (Figure 1). The development of a new platform for anti-TNF therapy is proposed in stages. In the first stage, patients with LOR may benefit from introducing variability in dosages and administration times, including variable drug holidays within a pre-determined range with regulatory approval. In the second stage, patient-tailored algorithms based on quantifying variability signatures that are directly or indirectly related to the underlying chronic inflammatory state and to the response to the anti-TNFs, including patients' variability patterns, will be applied.

In summary, the complexity of the immune response to anti-TNF mAbs induces compensatory adaptation at several cellular levels that jeopardize the response, resulting in primary or secondary failure. Introducing patient-tailored variability to drug administration may provide a method for reducing the LOR in such patients. The results of ongoing studies implementing these concepts using patient-tailored–based algorithms will shed light on some of the mechanisms involved in immune adaptation to anti-TNFs and may provide a means of improving the response to these drugs.

## Author Contributions

All authors listed have made a substantial, direct and intellectual contribution to the work, and approved it for publication.

### Conflict of Interest

YI is the founder of Oberon Sciences and is a consultant for Teva, ENZO, Protalix, Betalin Therapeutics, Immuron, SciM, Natural Shield, Oberon Sciences, Tiziana Pharma, Plantylight, and Exalenz Bioscience. The remaining author declares that the research was conducted in the absence of any commercial or financial relationships that could be construed as a potential conflict of interest.

## Abbreviations

mAbs: monoclonal antibodies
RA: rheumatoid arthritis
IBD: inflammatory bowel disease
DAS: disease activity score
EULAR: European League Against Rheumatism
ETA: etanercept
IFX: infliximab
ADM: adalimumab
LOR: loss of response
ADAbs: anti-drug antibodies
SNPs: single-nucleotide polymorphisms
GWAS: genome-wide association study
MTX: methotrexate
CD: Crohn's disease
ED: elemental diet
tmTNF: transmembrane tumor necrosis factor
TNFR: TNF receptor
FcRn: neonatal Fc receptor
AS: ankylosing spondylitis.

## References

[1] Taylor PC Feldmann M. Anti-TNF biologic agents: still the therapy of choice for rheumatoid arthritis. Nat Rev Rheumatol. (2009) 5:578–82. 10.1038/nrrheum.2009.181 19798034

[2] Rutgeerts P Van Assche G Vermeire S. Optimizing anti-TNF treatment in inflammatory bowel disease. Gastroenterology. (2004) 126:1593–610. 10.1053/j.gastro.2004.02.070 15168370

[3] Ha C Ullman TA Siegel CA Kornbluth A Patients enrolled in randomized controlled trials do not represent the inflammatory bowel disease patient population. Clin Gastroenterol Hepatol. (2012) 10:1002–7; quiz:e1078. 10.1016/j.cgh.2012.02.004 22343692

[4] Finckh A Simard JF Gabay C Guerne PA physicians S. Evidence for differential acquired drug resistance to anti-tumour necrosis factor agents in rheumatoid arthritis. Ann Rheum Dis. (2006) 65:746–52. 10.1136/ard.2005.045062 16339288 PMC1798167

[5] Kalden JR Schulze-Koops H. Immunogenicity and loss of response to TNF inhibitors: implications for rheumatoid arthritis treatment. Nat Rev Rheumatol. (2017) 13:707–18. 10.1038/nrrheum.2017.187 29158574

[6] Kremer JM Russell AS Emery P Abud-Mendoza C Szechinski J Westhovens R Li T. Long-term safety, efficacy and inhibition of radiographic progression with abatacept treatment in patients with rheumatoid arthritis and an inadequate response to methotrexate: 3-year results from the AIM trial. Ann Rheum Dis. (2011) 70:1826–30. 10.1136/ard.2010.139345 21893583 PMC3171107

[7] Sfikakis PP. The first decade of biologic TNF antagonists in clinical practice: lessons learned, unresolved issues and future directions. Curr Dir Autoimmun. (2010) 11:180–210. 10.1159/000289205 20173395

[8] Gottenberg JE Brocq O Perdriger A Lassoued S Berthelot JM Wendling D. Non-TNF-targeted biologic vs a second anti-TNF drug to treat rheumatoid arthritis in patients with insufficient response to a first anti-TNF drug: a randomized clinical trial. JAMA. (2016) 316:1172–80. 10.1001/jama.2016.13512 27654603

[9] Ceccarelli F Massafra U Perricone C Idolazzi L Giacomelli R Tirri R. Anti-TNF treatment response in rheumatoid arthritis patients with moderate disease activity: a prospective observational multicentre study (MODERATE). Clin Exp Rheumatol. (2017) 35:24–32. 27974105

[10] Weyand CM Goronzy JJ. Ectopic germinal center formation in rheumatoid synovitis. Ann N Y Acad Sci. (2003) 987:140–9. 10.1111/j.1749-6632.2003.tb06042.x 12727633

[11] Weyand CM Kang YM Kurtin PJ Goronzy JJ. The power of the third dimension: tissue architecture and autoimmunity in rheumatoid arthritis. Curr Opin Rheumatol. (2003) 15:259–66. 10.1097/00002281-200305000-00013 12707579

[12] Navarro Coy NC Brown S Bosworth A Davies CT Emery P Everett CC The ‘Switch' study protocol: a randomised-controlled trial of switching to an alternative tumour-necrosis factor (TNF)-inhibitor drug or abatacept or rituximab in patients with rheumatoid arthritis who have failed an initial TNF-inhibitor drug. BMC Musculoskelet Disord. (2014) 15:452 10.1186/1471-2474-15-452 25539805 PMC4391115

[13] Fransen J van Riel PL. The Disease Activity Score and the EULAR response criteria. Clin Exp Rheumatol. (2005) 23:S93–9. 10.1016/j.rdc.2009.10.001 16273792

[14] Fafa BP Louzada-Junior P Titton DC Zandonade E Ranza R Laurindo I. Drug survival and causes of discontinuation of the first anti-TNF in ankylosing spondylitis compared with rheumatoid arthritis: analysis from BIOBADABRASIL. Clin Rheumatol. (2015) 34:921–7. 10.1007/s10067-015-2929-7 25851594

[15] Souto A Maneiro JR Gomez-Reino JJ. Rate of discontinuation and drug survival of biologic therapies in rheumatoid arthritis: a systematic review and meta-analysis of drug registries and health care databases. Rheumatology. (2016) 55:523–34. 10.1093/rheumatology/kev374 26490106

[16] Biggioggero M Favalli EG. Ten-year drug survival of anti-TNF agents in the treatment of inflammatory arthritides. Drug Dev Res. (2014) 75(Suppl. 1):S38–41. 10.1002/ddr.21192 25381973

[17] Hanauer SB Feagan BG Lichtenstein GR Mayer LF Schreiber S Colombel JF. Maintenance infliximab for Crohn's disease: the ACCENT I randomised trial. Lancet. (2002) 359:1541–9. 10.1016/S0140-6736(02)08512-4 12047962

[18] Sands BE Anderson FH Bernstein CN Chey WY Feagan BG Fedorak RN. Infliximab maintenance therapy for fistulizing Crohn's disease. N Engl J Med. (2004) 350:876–85. 10.1056/NEJMoa030815 14985485

[19] D'Haens GR Panaccione R Higgins PD Vermeire S Gassull M Chowers Y. The London position statement of the world congress of gastroenterology on biological therapy for IBD with the European Crohn's and Colitis Organization: when to start, when to stop, which drug to choose, and how to predict response? Am J Gastroenterol. (2011) 106:199–212; quiz: 213. 10.1038/ajg.2010.392 21045814

[20] Allez M Karmiris K Louis E Van Assche G Ben-Horin S Klein A. Report of the ECCO pathogenesis workshop on anti-TNF therapy failures in inflammatory bowel diseases: definitions, frequency and pharmacological aspects. J Crohns Colitis. (2010) 4:355–66. 10.1016/j.crohns.2010.04.004 21122530

[21] Ben-Horin S Kopylov U Chowers Y. Optimizing anti-TNF treatments in inflammatory bowel disease. Autoimmun Rev. (2014) 13:24–30. 10.1016/j.autrev.2013.06.002 23792214

[22] Gisbert JP Panes J. Loss of response and requirement of infliximab dose intensification in Crohn's disease: a review. Am J Gastroenterol. (2009) 104:760–67. 10.1038/ajg.2008.88 19174781

[23] Billioud V Sandborn WJ Peyrin-Biroulet L. Loss of response and need for adalimumab dose intensification in Crohn's disease: a systematic review. Am J Gastroenterol. (2011) 106:674–84. 10.1038/ajg.2011.60 21407178

[24] Ma C Huang V Fedorak DK Kroeker KI Dieleman LA Halloran BP Crohn's disease outpatients treated with adalimumab have an earlier secondary loss of response and requirement for dose escalation compared to infliximab: a real life cohort study J Crohns Colitis. (2014) 8:1454–63. 10.1016/j.crohns.2014.05.007 24947334

[25] Qiu Y Chen BL Mao R Zhang SH He Y Zeng ZR. Systematic review with meta-analysis: loss of response and requirement of anti-TNFalpha dose intensification in Crohn's disease. J Gastroenterol. (2017) 52:535–54. 10.1007/s00535-017-1324-3 28275925

[26] Roblin X Rinaudo M Del Tedesco E Phelip JM Genin C Peyrin-Biroulet L. Development of an algorithm incorporating pharmacokinetics of adalimumab in inflammatory bowel diseases. Am J Gastroenterol. (2014) 109:1250–6. 10.1038/ajg.2014.146 24913041

[27] Chowers Y Sturm A Sans M Papadakis K Gazouli M Harbord M. Report of the ECCO workshop on anti-TNF therapy failures in inflammatory bowel diseases: biological roles and effects of TNF and TNF antagonists. J Crohns Colitis. (2010) 4:367–76. 10.1016/j.crohns.2010.05.011 21122531

[28] Keizer RJ Huitema AD Schellens JH Beijnen JH. Clinical pharmacokinetics of therapeutic monoclonal antibodies. Clin Pharmacokinet. (2010) 49:493–507. 10.2165/11531280-000000000-00000 20608753

[29] Tabrizi MA Tseng CM Roskos LK. Elimination mechanisms of therapeutic monoclonal antibodies. Drug Discov Tdy. (2006) 11:81–8. 10.1016/S1359-6446(05)03638-X 16478695

[30] Ordas I Feagan BG Sandborn WJ. Therapeutic drug monitoring of tumor necrosis factor antagonists in inflammatory bowel disease. Clin Gastroenterol Hepatol. (2012) 10:1079–87; quiz: e1085. 10.1016/j.cgh.2012.06.032 22813440

[31] Pink AE Fonia A Allen MH Smith CH Barker JN. Antinuclear antibodies associate with loss of response to antitumour necrosis factor-alpha therapy in psoriasis: a retrospective, observational study. Br J Dermatol. (2010) 162:780–5. 10.1111/j.1365-2133.2009.09563.x 19863499

[32] Luther J Gala M Patel SJ Dave M Borren N Xavier RJ Loss of response to anti-tumor necrosis factor alpha therapy in Crohn's disease is not associated with emergence of novel inflammatory pathways. Dig Dis Sci. (2018) 63:738–45. 10.1007/s10620-018-4932-8 29372477 PMC6152907

[33] Haberman Y Tickle TL Dexheimer PJ Kim MO Tang D Karns R. Pediatric Crohn disease patients exhibit specific ileal transcriptome and microbiome signature. J Clin Invest. (2014) 124:3617–33. 10.1172/JCI75436 25003194 PMC4109533

[34] Kong JY Bundell C Pawlik J Hollingsworth P Forbes G. Low trough serum infliximab and antibodies to infliximab in smokers. Inflamm Bowel Dis. (2013) 19:E35–6. 10.1002/ibd.22928 22345044

[35] Srinivasan A Vasudevan A McFarlane A Sparrow MP Gibson PR Van Langenberg DR. Anti-TNF re-induction is as effective, simpler, and cheaper compared with dose interval shortening for secondary loss of response in Crohn's disease. J Crohns Colitis. (2018) 12:280–8. 10.1093/ecco-jcc/jjx144 29077839

[36] Scaldaferri F D'Ambrosio D Holleran G Poscia A Petito V Lopetuso L. Body mass index influences infliximab post-infusion levels and correlates with prospective loss of response to the drug in a cohort of inflammatory bowel disease patients under maintenance therapy with Infliximab. PLoS ONE. (2017) 12:e0186575. 10.1371/journal.pone.0186575 29073159 PMC5657978

[37] Moran GW Dubeau MF Kaplan GG Yang H Seow CH Fedorak RN. Phenotypic features of Crohn's disease associated with failure of medical treatment. Clin Gastroenterol Hepatol. (2014) 12:434–42.e1. 10.1016/j.cgh.2013.08.026 23978351

[38] Fasanmade AA Adedokun OJ Ford J Hernandez D Johanns J Hu C. Population pharmacokinetic analysis of infliximab in patients with ulcerative colitis. Eur J Clin Pharmacol. (2009) 65:1211–28. 10.1007/s00228-009-0718-4 19756557 PMC2778780

[39] Fasanmade AA Adedokun OJ Blank M Zhou H Davis HM. Pharmacokinetic properties of infliximab in children and adults with Crohn's disease: a retrospective analysis of data from 2 phase III clinical trials. Clin Ther. (2011) 33:946–964. 10.1016/j.clinthera.2011.06.002 21741088

[40] Brandse JF van den Brink GR Wildenberg ME van der Kleij D Rispens T Jansen JM. Loss of infliximab into feces is associated with lack of response to therapy in patients with severe ulcerative colitis. Gastroenterology. (2015) 149:350–5.e2. 10.1053/j.gastro.2015.04.016 25917786

[41] Hanauer SB Sandborn WJ Rutgeerts P Fedorak RN Lukas M MacIntosh D. Human anti-tumor necrosis factor monoclonal antibody (adalimumab) in Crohn's disease: the CLASSIC-I trial. Gastroenterology. (2006) 130:323–33; quiz: 591. 10.1053/j.gastro.2005.11.030 16472588

[42] Colombel JF Sandborn WJ Reinisch W Mantzaris GJ Kornbluth A Rachmilewitz D Infliximab, azathioprine, or combination therapy for Crohn's disease. N Engl J Med. (2010) 362:1383–95. 10.1056/NEJMoa0904492 20393175

[43] Cuchacovich M Bueno D Carvajal R Bravo N Aguillon JC Catalan D. Clinical parameters and biomarkers for anti-TNF treatment prognosis in rheumatoid arthritis patients. Clin Rheumatol. (2014) 33:1707–14. 10.1007/s10067-014-2756-2 25085274

[44] Hyrich KL Watson KD Silman AJ Symmons DP British Society for Rheumatology Biologics Register. Predictors of response to anti-TNF-alpha therapy among patients with rheumatoid arthritis: results from the British Society for Rheumatology Biologics Register. Rheumatology. (2006) 45:1558–65. 10.1093/rheumatology/kel149 16705046

[45] Mugnier B Balandraud N Darque A Roudier C Roudier J Reviron D. Polymorphism at position−308 of the tumor necrosis factor alpha gene influences outcome of infliximab therapy in rheumatoid arthritis. Arthritis Rheum. (2003) 48:1849–52. 10.1002/art.11168 12847678

[46] Viatte S Plant D Han B Fu B Yarwood A Thomson W. Association of HLA-DRB1 haplotypes with rheumatoid arthritis severity, mortality, and treatment response. JAMA. (2015) 313:1645–56. 10.1001/jama.2015.3435 25919528 PMC4928097

[47] Montes A Perez-Pampin E Navarro-Sarabia F Moreira V de la Serna AR Magallares B. Rheumatoid arthritis response to treatment across IgG1 allotype - anti-TNF incompatibility: a case-only study. Arthritis Res Ther. (2015) 17:63. 10.1186/s13075-015-0571-z 25885039 PMC4411723

[48] Canet LM Filipescu I Caliz R Lupianez CB Canhao H Escudero A. Genetic variants within the TNFRSF1B gene and susceptibility to rheumatoid arthritis and response to anti-TNF drugs: a multicenter study. Pharmacogenet Genomics. (2015) 25:323–33. 10.1097/FPC.0000000000000140 25850964

[49] Canet LM Sanchez-Maldonado JM Caliz R Rodriguez-Ramos A Lupianez CB Canhao H. Polymorphisms at phase I-metabolizing enzyme and hormone receptor loci influence the response to anti-TNF therapy in rheumatoid arthritis patients. Pharmacogenomics J. (2019) 19:83–96. 10.1038/s41397-018-0057-x 30287909

[50] Bek S Bojesen AB Nielsen JV Sode J Bank S Vogel U. Systematic review and meta-analysis: pharmacogenetics of anti-TNF treatment response in rheumatoid arthritis. Pharmacogenomics J. (2017) 17:403–11. 10.1038/tpj.2017.26 28607508 PMC5637244

[51] Julia A Fernandez-Nebro A Blanco F Ortiz A Canete JD Maymo J. A genome-wide association study identifies a new locus associated with the response to anti-TNF therapy in rheumatoid arthritis. Pharmacogenomics J. (2016) 16:147–50. 10.1038/tpj.2015.31 25896534

[52] Borras-Blasco J Navarro Ruiz A. Dose modification of anti-TNF in rheumatoid arthritis and estimated economical impact: a review of observational studies. Expert Rev Pharmacoecon Outcomes Res. (2015) 15:71–9. 10.1586/14737167.2015.967219 25555555

[53] Anderson PJ. Tumor necrosis factor inhibitors: clinical implications of their different immunogenicity profiles. Semin Arthritis Rheum. (2005) 34:19–22. 10.1016/j.semarthrit.2005.01.005 15852250

[54] Mok CC Tsai WC Chen DY Wei JC. Immunogenicity of anti-TNF biologic agents in the treatment of rheumatoid arthritis. Expert Opin Biol Ther. (2016) 16:201–11. 10.1517/14712598.2016.1118457 26560845

[55] Tracey D Klareskog L Sasso EH Salfeld JG Tak PP. Tumor necrosis factor antagonist mechanisms of action: a comprehensive review. Pharmacol Ther. (2008) 117:244–79. 10.1016/j.pharmthera.2007.10.001 18155297

[56] Radstake TR Svenson M Eijsbouts AM van den Hoogen FH Enevold C van Riel PL. Formation of antibodies against infliximab and adalimumab strongly correlates with functional drug levels and clinical responses in rheumatoid arthritis. Ann Rheum Dis. (2009) 68:1739–45. 10.1136/ard.2008.092833 19019895

[57] Bartelds GM Wijbrandts CA Nurmohamed MT Stapel S Lems WF Aarden L. Clinical response to adalimumab: relationship to anti-adalimumab antibodies and serum adalimumab concentrations in rheumatoid arthritis. Ann Rheum Dis. (2007) 66:921–6. 10.1136/ard.2006.065615 17301106 PMC1955110

[58] Hart MH de Vrieze H Wouters D Wolbink GJ Killestein J de Groot ER. Differential effect of drug interference in immunogenicity assays. J Immunol Methods. (2011) 372:196–203. 10.1016/j.jim.2011.07.019 21824477

[59] Pouw MF Krieckaert CL Nurmohamed MT van der Kleij D Aarden L Rispens T. Key findings towards optimising adalimumab treatment: the concentration-effect curve. Ann Rheum Dis. (2015) 74:513–8. 10.1136/annrheumdis-2013-204172 24326008

[60] Wu C Wang S Xian P Yang L Chen Y Mo X. Effect of anti-TNF antibodies on clinical response in rheumatoid arthritis patients: a meta-analysis. Biomed Res Int. (2016) 2016:7185708. 10.1155/2016/7185708 27556040 PMC4983315

[61] Chen DY Chen YM Hsieh TY Hung WT Hsieh CW Chen HH. Drug trough levels predict therapeutic responses to dose reduction of adalimumab for rheumatoid arthritis patients during 24 weeks of follow-up. Rheumatology. (2016) 55:143–8. 10.1093/rheumatology/kev298 26324949

[62] Agarwal SK Glass RJ Shadick NA Coblyn JS Anderson RJ Maher NE. Predictors of discontinuation of tumor necrosis factor inhibitors in patients with rheumatoid arthritis. J Rheumatol. (2008) 35:1737–44. 18634159 PMC2756035

[63] Anecchino C Fanizza C Marino V Romero M Group DS. Drug outcome survey to evaluate anti-TNF treatment in rheumatoid arthritis: an Italian observational study (the DOSE study). Clin Exp Rheumatol. (2015) 33:779–87. 26575614

[64] Potter C Hyrich KL Tracey A Lunt M Plant D Symmons DP. Association of rheumatoid factor and anti-cyclic citrullinated peptide positivity, but not carriage of shared epitope or PTPN22 susceptibility variants, with anti-tumour necrosis factor response in rheumatoid arthritis. Ann Rheum Dis. (2009) 68:69–74. 10.1136/ard.2007.084715 18375541 PMC2596303

[65] Eng GP Bouchelouche P Bartels EM Bliddal H Bendtzen K Stoltenberg M. Anti-drug antibodies, drug levels, interleukin-6 and soluble TNF receptors in rheumatoid arthritis patients during the first 6 months of treatment with adalimumab or infliximab: a descriptive cohort study. PLoS ONE. (2016) 11:e0162316. 10.1371/journal.pone.0162316 27606615 PMC5016088

[66] Tweehuysen L den Broeder N van Herwaarden N Joosten LAB van Lent PL Vogl T. Predictive value of serum calprotectin (S100A8/A9) for clinical response after starting or tapering anti-TNF treatment in patients with rheumatoid arthritis. RMD Open. (2018) 4:e000654. 10.1136/rmdopen-2018-000654 29657832 PMC5892754

[67] Garces S Antunes M Benito-Garcia E da Silva JC Aarden L Demengeot J. A preliminary algorithm introducing immunogenicity assessment in the management of patients with RA receiving tumour necrosis factor inhibitor therapies. Ann Rheum Dis. (2014) 73:1138–43. 10.1136/annrheumdis-2013-203296 23666932

[68] Garces S Demengeot J Benito-Garcia E. The immunogenicity of anti-TNF therapy in immune-mediated inflammatory diseases: a systematic review of the literature with a meta-analysis. Ann Rheum Dis. (2013) 72:1947–55. 10.1136/annrheumdis-2012-202220 23223420

[69] Vogelzang EH Pouw MF Nurmohamed M Kneepkens EL Rispens T Wolbink GJ. Adalimumab trough concentrations in patients with rheumatoid arthritis and psoriatic arthritis treated with concomitant disease-modifying antirheumatic drugs. Ann Rheum Dis. (2015) 74:474–5. 10.1136/annrheumdis-2014-206588 25433018

[70] Levy RA Guzman R Castaneda-Hernandez G Martinez-Vazquez M Damian G Cara C. Biology of anti-TNF agents in immune-mediated inflammatory diseases: therapeutic implications. Immunotherapy. (2016) 8:1427–36. 10.2217/imt-2016-0067 27737604

[71] Hyrich KL Lunt M Watson KD Symmons DP Silman AJ British Society for Rheumatology Biologics Register. Outcomes after switching from one anti-tumor necrosis factor alpha agent to a second anti-tumor necrosis factor alpha agent in patients with rheumatoid arthritis: results from a large UK national cohort study. Arthritis Rheum. (2007) 56:13–20. 10.1002/art.22331 17195186

[72] Du Pan SM Scherer A Gabay C Finckh A. Differential drug retention between anti-TNF agents and alternative biological agents after inadequate response to an anti-TNF agent in rheumatoid arthritis patients. Ann Rheum Dis. (2012) 71:997–9. 10.1136/annrheumdis-2011-200882 22294628

[73] Virkki LM Valleala H Takakubo Y Vuotila J Relas H Komulainen R. Outcomes of switching anti-TNF drugs in rheumatoid arthritis–a study based on observational data from the Finnish Register of Biological Treatment (ROB-FIN). Clin Rheumatol. (2011) 30:1447–54. 10.1007/s10067-011-1779-1 21644062

[74] Lequerre T Farran E Menard JF Kozyreff-Meurice M Vandhuick T Tharasse C. Switching from an anti-TNF monoclonal antibody to soluble TNF-receptor yields better results than vice versa: an observational retrospective study of 72 rheumatoid arthritis switchers. Joint Bone Spine. (2015) 82:330–7. 10.1016/j.jbspin.2015.01.021 25864942

[75] Soares MR Reis Neto ET Luz KR Ciconelli RM Pinheiro MM Switching between anti-TNF-alpha agents does not improve functional capacity in patients with long-standing and active rheumatoid arthritis. Rev Bras Reumatol. (2012) 52:9–15. 10.1590/S0482-50042012000100002 22286641

[76] Sandborn WJ. Preventing antibodies to infliximab in patients with Crohn's disease: optimize not immunize. Gastroenterology. (2003) 124:1140–5. 10.1053/gast.2003.50182 12671907

[77] Steenholdt C Brynskov J Thomsen OO Munck LK Fallingborg J Christensen LA. Individualised therapy is more cost-effective than dose intensification in patients with Crohn's disease who lose response to anti-TNF treatment: a randomised, controlled trial. Gut. (2014) 63:919–27. 10.1136/gutjnl-2013-305279 23878167

[78] Duveau N Nachury M Gerard R Branche J Maunoury V Boualit M. Adalimumab dose escalation is effective and well tolerated in Crohn's disease patients with secondary loss of response to adalimumab. Dig Liver Dis. (2017) 49:163–9. 10.1016/j.dld.2016.11.002 27899263

[79] Guidi L Pugliese D Panici Tonucci T Berrino A Tolusso B Basile M Therapeutic drug monitoring is more cost-effective than a clinically-based approach in the management of loss of response to infliximab in inflammatory bowel disease: an observational multi-centre study. J Crohns Colitis. (2018) 12:1079–88. 10.1093/ecco-jcc/jjy076 29860436

[80] Ben-Horin S Chowers Y. Tailoring anti-TNF therapy in IBD: drug levels and disease activity. Nat Rev Gastroenterol Hepatol. (2014) 11:243–55. 10.1038/nrgastro.2013.253 24393836

[81] Ding NS Hart A De Cruz P. Systematic review: predicting and optimising response to anti-TNF therapy in Crohn's disease - algorithm for practical management. Aliment Pharmacol Ther. (2016) 43:30–51. 10.1111/apt.13445 26515897

[82] Vande Casteele N Gils A Singh S Ohrmund L Hauenstein S Rutgeerts P. Antibody response to infliximab and its impact on pharmacokinetics can be transient. Am J Gastroenterol. (2013) 108:962–71. 10.1038/ajg.2013.12 23419382

[83] Hibi T Sakuraba A Watanabe M Motoya S Ito H Motegi K. Retrieval of serum infliximab level by shortening the maintenance infusion interval is correlated with clinical efficacy in Crohn's disease. Inflamm Bowel Dis. (2012) 18:1480–7. 10.1002/ibd.21886 21987418

[84] Afif W Loftus EV Jr Faubion WA Kane SV Bruining DH Hanson KA. Clinical utility of measuring infliximab and human anti-chimeric antibody concentrations in patients with inflammatory bowel disease. Am J Gastroenterol. (2010) 105:1133–9. 10.1038/ajg.2010.9 20145610 PMC6937708

[85] Yanai H Lichtenstein L Assa A Mazor Y Weiss B Levine A Levels of drug and antidrug antibodies are associated with outcome of interventions after loss of response to infliximab or adalimumab. Clin Gastroenterol Hepatol. (2015) 13:522–30.e22. 10.1016/j.cgh.2014.07.029 25066837

[86] Bartelds GM Krieckaert CL Nurmohamed MT van Schouwenburg PA Lems WF Twisk JW. Development of antidrug antibodies against adalimumab and association with disease activity and treatment failure during long-term follow-up. JAMA. (2011) 305:1460–8. 10.1001/jama.2011.406 21486979

[87] Pariente B Pineton de Chambrun G Krzysiek R Desroches M Louis G De Cassan C Trough levels and antibodies to infliximab may not predict response to intensification of infliximab therapy in patients with inflammatory bowel disease. Inflamm Bowel Dis. (2012) 18:1199–206. 10.1002/ibd.21839 22127789

[88] Steenholdt C Bendtzen K Brynskov J Thomsen OO Munck LK Christensen LA Changes in serum trough levels of infliximab during treatment intensification but not in anti-infliximab antibody detection are associated with clinical outcomes after therapeutic failure in Crohn's disease. J Crohns Colitis. (2015) 9:238–45. 10.1093/ecco-jcc/jjv004 25576753

[89] Mosli MH Sandborn WJ Kim RB Khanna R Al-Judaibi B Feagan BG. Toward a personalized medicine approach to the management of inflammatory bowel disease. Am J Gastroenterol. (2014) 109:994–1004. 10.1038/ajg.2014.110 24842338

[90] Colombel JF Feagan BG Sandborn WJ Van Assche G Robinson AM. Therapeutic drug monitoring of biologics for inflammatory bowel disease. Inflamm Bowel Dis. (2012) 18:349–58. 10.1002/ibd.21831 22021134

[91] Vermeire S Noman M Van Assche G Baert F D'Haens G Rutgeerts P. Effectiveness of concomitant immunosuppressive therapy in suppressing the formation of antibodies to infliximab in Crohn's disease. Gut. (2007) 56:1226–31. 10.1136/gut.2006.099978 17229796 PMC1954977

[92] Sokol H Seksik P Carrat F Nion-Larmurier I Vienne A Beaugerie L. Usefulness of co-treatment with immunomodulators in patients with inflammatory bowel disease treated with scheduled infliximab maintenance therapy. Gut. (2010) 59:1363–8. 10.1136/gut.2010.212712 20587545

[93] Ben-Horin S Waterman M Kopylov U Yavzori M Picard O Fudim E. Addition of an immunomodulator to infliximab therapy eliminates antidrug antibodies in serum and restores clinical response of patients with inflammatory bowel disease. Clin Gastroenterol Hepatol. (2013) 11:444–7. 10.1016/j.cgh.2012.10.020 23103905

[94] Jones JL Kaplan GG Peyrin-Biroulet L Baidoo L Devlin S Melmed GY. Effects of concomitant immunomodulator therapy on efficacy and safety of anti-tumor necrosis factor therapy for Crohn's disease: a meta-analysis of placebo-controlled trials. Clin Gastroenterol Hepatol. (2015) 13:2233–40.e1–2; quiz: e177–8. 10.1016/j.cgh.2015.06.034 26142167

[95] Varma P Rajadurai AS Holt DQ Devonshire DA Desmond CP Swan MP. Immunomodulator use does not prevent first loss of response to anti-TNF therapy in inflammatory bowel disease: long term outcomes in a real-world cohort. Intern Med J. (2018) 49:685–810. 10.1111/imj.14150 30381884

[96] Peeters H Louis E Baert F Dewit O Coche JC Ferrante M. Efficacy of switching to infliximab in patients with Crohn's disease with loss of response to adalimumab. Acta Gastroenterol Belg. (2018) 81:15–21. 29562373

[97] Ungar B Kopylov U Engel T Yavzori M Fudim E Picard O Addition of an immunomodulator can reverse antibody formation and loss of response in patients treated with adalimumab. Aliment Pharmacol Ther. (2017) 45:276–82. 10.1111/apt.13862 27862102

[98] Sugita N Watanabe K Kamata N Yukawa T Otani K Hosomi S. Efficacy of a concomitant elemental diet to reduce the loss of response to adalimumab in patients with intractable Crohn's disease. J Gastroenterol Hepatol. (2018) 33:631–7. 10.1111/jgh.13969 28857255

[99] Ducourau E Mulleman D Paintaud G Miow Lin DC Lauferon F Ternant D. Antibodies toward infliximab are associated with low infliximab concentration at treatment initiation and poor infliximab maintenance in rheumatic diseases. Arthritis Res Ther. (2011) 13:R105. 10.1186/ar3386 21708018 PMC3218920

[100] Rutgeerts P Feagan BG Lichtenstein GR Mayer LF Schreiber S Colombel JF. Comparison of scheduled and episodic treatment strategies of infliximab in Crohn's disease. Gastroenterology. (2004) 126:402–13. 10.1053/j.gastro.2003.11.014 14762776

[101] Kollias G Kontoyiannis D. Role of TNF/TNFR in autoimmunity: specific TNF receptor blockade may be advantageous to anti-TNF treatments. Cytokine Growth Factor Rev. (2002) 13:315–21. 10.1016/S1359-6101(02)00019-9 12220546

[102] Talotta R Berzi A Atzeni F Batticciotto A Clerici M Sarzi-Puttini P. Paradoxical expansion of Th1 and Th17 lymphocytes in rheumatoid arthritis following infliximab treatment: a possible explanation for a lack of clinical response. J Clin Immunol. (2015) 35:550–7. 10.1007/s10875-015-0182-0 26271387

[103] Shovman O Tamar S Amital H Watad A Shoenfeld Y. Diverse patterns of anti-TNF-alpha-induced lupus: case series and review of the literature. Clin Rheumatol. (2018) 37:563–8. 10.1007/s10067-017-3884-2 29063464

[104] Honda Y Otsuka A Egawa G Inoue Y Kuzuya A Takahashi R. Multiple neurological abnormalities, including pontine hemorrhage, multiple sclerosis and aseptic meningitis, during anti-TNF-alpha therapy in psoriatic arthritis. Eur J Dermatol. (2015) 25:487–8. 10.1684/ejd.2015.2558 26693636

[105] Wang M Meng X Tsai B Wang JF Turrentine M Brown JW. Preconditioning up-regulates the soluble TNF receptor I response to endotoxin. J Surg Res. (2004) 121:20–4. 10.1016/j.jss.2004.02.017 15313370

[106] Fairfax BP Davenport EE Makino S Hill AV Vannberg FO Knight JC. A common haplotype of the TNF receptor 2 gene modulates endotoxin tolerance. J Immunol. (2011) 186:3058–65. 10.4049/jimmunol.1001791 21282507 PMC3211060

[107] Deora A Hegde S Lee J Choi CH Chang Q Lee C. Transmembrane TNF-dependent uptake of anti-TNF antibodies. MAbs. (2017) 9:680–95. 10.1080/19420862.2017.1304869 28323513 PMC5419086

[108] Takahashi N Brouckaert P Bemelmans MH Buurman WA Fiers W Mechanism of induction of tolerance to tumour necrosis factor (TNF): no involvement of modulators of TNF bioavailability or receptor binding. Cytokine. (1994) 6:235–42. 10.1016/1043-4666(94)90018-3 8054478

[109] Ilan Y. Generating randomness: making the most out of disordering a false order into a real one. J Transl Med. (2019) 17:49. 10.1186/s12967-019-1798-2 30777074 PMC6379992

[110] Ilan Y. Randomness in microtubule dynamics: an error that requires correction or an inherent plasticity required for normal cellular function? Cell Biol Int. (2019) 7:739–48. 10.1002/cbin.11157 31042006

[111] Singh N Moneghetti KJ Christle JW Hadley D Froelicher V Plews D. Heart rate variability: an old metric with new meaning in the era of using mhealth technologies for health and exercise training guidance. part two: prognosis and training. Arrhythm Electrophysiol Rev. (2018) 7:247–55. 10.15420/aer.2018.30.2 30588312 PMC6304793

[112] Lees T Shad-Kaneez F Simpson AM Nassif NT Lin Y Lal S. Heart rate variability as a biomarker for predicting stroke, post-stroke complications and functionality. Biomark Insights. (2018) 13:1177271918786931. 10.1177/1177271918786931 30038486 PMC6052496

[113] Herssens N Verbecque E Hallemans A Vereeck L Van Rompaey V Saeys W. Do spatiotemporal parameters and gait variability differ across the lifespan of healthy adults? A systematic review. Gait Posture. (2018) 64:181–90. 10.1016/j.gaitpost.2018.06.012 29929161

[114] Henriques T Munshi MN Segal AR Costa MD Goldberger AL. “Glucose-at-a-Glance”: new method to visualize the dynamics of continuous glucose monitoring data. J Diabetes Sci Technol. (2014) 8:299–306. 10.1177/1932296814524095 24876582 PMC4455408

[115] Tosato F Bernardi D Sanzari MC Pantano G Plebani M. Biological variability of lymphocyte subsets of human adults' blood. Clin Chim Acta. (2013) 424:159–63. 10.1016/j.cca.2013.06.001 23770423

[116] Mitchell S Roy K Zangle TA Hoffmann A. Nongenetic origins of cell-to-cell variability in B lymphocyte proliferation. Proc Natl Acad Sci USA. (2018) 115:E2888–E2897. 10.1073/pnas.1715639115 29514960 PMC5866559

[117] Fuchs A Gliwinski M Grageda N Spiering R Abbas AK Appel S. Minimum information about T regulatory cells: a step toward reproducibility and standardization. Front Immunol. (2017) 8:1844. 10.3389/fimmu.2017.01844 29379498 PMC5775516

[118] Liebers V Kendzia B Stubel H Borowitzki G Gering V Monse C. Cell activation and cytokine release *ex vivo*: estimation of reproducibility of the whole-blood assay with fresh human blood. Adv Exp Med Biol. (2018) 1108:25–36. 10.1007/5584_2018_225 29931563

[119] Leino AD King EC Jiang W Vinks AA Klawitter J Christians U. Assessment of tacrolimus intrapatient variability in stable adherent transplant recipients: Establishing baseline values. Am J Transplant. (2018) 19:1410–20. 10.1111/ajt.15199 30506623

[120] Gueta I Markovits N Yarden-Bilavsky H Raichlin E Freimark D Lavee J. High tacrolimus trough level variability is associated with rejections after heart transplant. Am J Transplant. (2018) 18:2571–8. 10.1111/ajt.15016 29989311

[121] Gueta I Markovits N Yarden-Bilavsky H Raichlin E Freimark D Lavee J. Intrapatient variability in tacrolimus trough levels after solid organ transplantation varies at different postoperative time periods. Am J Transplant. (2018) 19:611–9. 10.1111/ajt.15134 30282112

[122] Del Bello A Congy-Jolivet N Danjoux M Muscari F Lavayssiere L Esposito L. High tacrolimus intra-patient variability is associated with graft rejection, and de novo donor-specific antibodies occurrence after liver transplantation. World J Gastroenterol. (2018) 24:1795–802. 10.3748/wjg.v24.i16.1795 29713132 PMC5922997

[123] Elgart V Lin JR Loscalzo J. Determinants of drug-target interactions at the single cell level. PLoS Comput Biol. (2018) 14:e1006601. 10.1371/journal.pcbi.1006601 30571695 PMC6319770

[124] Contin M Alberghini L Candela C Benini G Riva R. Intrapatient variation in antiepileptic drug plasma concentration after generic substitution vs stable brand-name drug regimens. Epilepsy Res. (2016) 122:79–83. 10.1016/j.eplepsyres.2016.02.012 26987080

[125] Rensing N Han L Wong M. Intermittent dosing of rapamycin maintains antiepileptogenic effects in a mouse model of tuberous sclerosis complex. Epilepsia. (2015) 56:1088–97. 10.1111/epi.13031 26122303 PMC4496285

[126] Ferriols-Lisart R Ferriols-Lisart F. Dose modifications of anti-TNF drugs in rheumatoid arthritis patients under real-world settings: a systematic review. Rheumatol Int. (2015) 35:1193–210. 10.1007/s00296-015-3222-4 25638015

[127] Pontes C Gratacos J Torres F Avendano C Sanz J Vallano A. Evaluation of dose reduction versus standard dosing for maintenance of remission in patients with spondyloarthritis and clinical remission with anti-TNF (REDES-TNF): study protocol for a randomized controlled trial. Trials. (2015) 16:370. 10.1186/s13063-015-0828-5 26289076 PMC4546086

[128] Inciarte-Mundo J Hernandez MV Rosario V Ruiz-Esquide V Cabrera-Villalba S Ramirez J. Reduction of biological agent dose in rheumatic diseases: descriptive analysis of 153 patients in clinical practice conditions. Reumatol Clin. (2014) 10:10–6. 10.1016/j.reumae.2013.11.005 23876791

[129] Den Broeder AA Creemers MC van Gestel AM van Riel PL. Dose titration using the Disease Activity Score (DAS28) in rheumatoid arthritis patients treated with anti-TNF-alpha. Rheumatology. (2002) 41:638–42. 10.1093/rheumatology/41.6.638 12048289

[130] Zavada J Uher M Sisol K Forejtova S Jarosova K Mann H. A tailored approach to reduce dose of anti-TNF drugs may be equally effective, but substantially less costly than standard dosing in patients with ankylosing spondylitis over 1 year: a propensity score-matched cohort study. Ann Rheum Dis. (2016) 75:96–102. 10.1136/annrheumdis-2014-205202 25165033

[131] Strik AB Mould S Mathôt D Ponsioen R van den Brande C Jansen J Dashboard driven vs. conventional dosing of infliximab in inflammatory bowel disease patients: the PRECISION trial. J Crohns Colitis. (2019) 13:S063 10.1093/ecco-jcc/jjy222.090

[132] de Lorenzo V Schmidt M. biological standards for the knowledge-based bioeconomy: what is at stake. Nat Biotechnol. (2018) 40:170–80. 10.1016/j.nbt.2017.05.001 28479235

[133] Gsponer J Babu MM The rules of disorder or why disorder rules. Prog Biophys Mol Biol. (2009) 99:94–103. 10.1016/j.pbiomolbio.2009.03.001 19344736

[134] Buckle AM Borg NA. Integrating experiment and theory to understand TCR-pMHC dynamics. Front Immunol. (2018) 9:2898. 10.3389/fimmu.2018.02898 30581442 PMC6293202

[135] Lodygin D Flugel A. Intravital real-time analysis of T-cell activation in health and disease. Cell Calcium. (2017) 64:118–129. 10.1016/j.ceca.2016.12.007 28126314

[136] Stergiou N Decker LM. Human movement variability, nonlinear dynamics, and pathology: is there a connection? Hum Mov Sci. (2011) 30:869–88. 10.1016/j.humov.2011.06.002 21802756 PMC3183280

[137] Ilan Y. Advanced tailored randomness: a novel approach for improving the efficacy of biological systems. J Comput Biol. (2019). 10.1089/cmb.2019.0231. [Epub ahead of print]. 31424268

[138] Ilan Y Why targeting the microbiome is not so successful: can randomness overcome the adaptation that occurs following gut manipulation? Clin Exp Gastroenterol. (2019) 12:209–17. 10.2147/CEG.S203823 31190948 PMC6514118

[139] Ilan Y. beta-Glycosphingolipids as mediators of both inflammation and immune tolerance: a manifestation of randomness in biological systems. Front Immunol. (2019) 10:1143. 10.3389/fimmu.2019.01143 31178868 PMC6538797

[140] Weiner WJ Koller WC Perlik S Nausieda PA Klawans HL. Drug holiday and management of Parkinson disease. Neurology. (1980) 30:1257–61. 10.1212/WNL.30.12.1257 7192805

[141] Toni T Tidor B. Combined model of intrinsic and extrinsic variability for computational network design with application to synthetic biology. PLoS Comput Biol. (2013) 9:e1002960. 10.1371/journal.pcbi.1002960 23555205 PMC3610654

[142] Orsini C Dankulov MM Colomer-de-Simon P Jamakovic A Mahadevan P Vahdat A. Quantifying randomness in real networks. Nat Commun. (2015) 6:8627. 10.1038/ncomms9627 26482121 PMC4667701

[143] Ghanjal A Motaqi M Arab Z Hatef B. Force variability in the short- and long-term type 2 diabetes mellitus. J Med Signals Sens. (2019) 9:50–8. 10.4103/jmss.JMSS_24_18 30967990 PMC6419562

